# An *EGFR* L858R mutation identified in 1862 Chinese NSCLC patients can be a promising neoantigen vaccine therapeutic strategy

**DOI:** 10.3389/fimmu.2022.1022598

**Published:** 2022-11-23

**Authors:** Jing Lin, Jun Liu, Shi-guang Hao, Bin Lan, Xiao-bin Zheng, Jia-ni Xiong, Ying-qian Zhang, Xuan Gao, Chuan-ben Chen, Ling Chen, Yu-fang Huang, Hong Luo, Yu-ting Yi, Xin Yi, Jian-ping Lu, Xiong-wei Zheng, Gang Chen, Xue-feng Wang, Yu Chen

**Affiliations:** ^1^ Department of Medical Oncology, Clinical Oncology School of Fujian Medical University, Fujian Cancer Hospital, Fuzhou, China; ^2^ Cancer Bio-immunotherapy Center, Clinical Oncology School of Fujian Medical University, Fujian Cancer Hospital, Fuzhou, China; ^3^ Geneplus-Beijing Institute, Beijing, China; ^4^ Fujian Provincial Key Laboratory of Tumor Biotherapy, Clinical Oncology School of Fujian Medical University, Fujian Cancer Hospital, Fuzhou, China; ^5^ State Key Laboratory of Microbial Resources, Institute of Microbiology, Chinese Academy of Sciences, Beijing, China; ^6^ Department of Translational Medicine, GenePlus- Shenzhen Clinical Laboratory, ShenZhen, China; ^7^ Department of Radiation Oncology, Clinical Oncology School of Fujian Medical University, Fujian Cancer Hospital, Fuzhou, China; ^8^ Department of Pathology, Clinical Oncology School of Fujian Medical University, Fujian Cancer Hospital, Fuzhou, China; ^9^ The Third Affiliated Hospital of Soochow University, Institutes for Translational Medicine, Soochow University, Suzhou, China

**Keywords:** *EGFR* L858R mutation, neoantigen vaccine, HLA A*33:03, immunological features, Chinese NSCLC

## Abstract

**Background:**

This study aimed to develop a vaccine that targets mutation-derived neoantigen in Chinese non-small-cell lung cancer (NSCLC).

**Methods:**

A cohort of 1862 Chinese NSCLC patients who underwent targeted sequencing with a 1021-gene panel was investigated. HLA typing was done using OptiType v1.0 and neoantigens were predicted by netMHCpan v4.0. HLA LOH was inferred using the lohhla algorithm and TMB were quantified by counting the total number of non-synonymous ones based on our panel data. CIBERSORT was utilized to estimate the TME in different *EGFR* mutant subtype by using TCGA data.

**Results:**

HLA-A*11:01(42.59%) was the top one allele and HLA-A*33:03(12.94%) ranked 12th. *EGFR* L858R (22.61%) was the most prevalent gene variant. The binding affinity (IC50 MT = 22.9 nM) and shared frequency (2.93%) of *EGFR* L858R in combination with HLA-A*33:03 were optimal. In a subsequent further analysis on immunological features of *EGFR* mutant subtypes, 63.1% HLA loss of heterozygosity LOH (HLA LOH) and 0.37% (7 of 1862) B2M aberrations were found in our population, both had no significant association with *EGFR* mutant subtypes suggesting that the process of antigen presentation involved HLA LOH and B2M mechanisms in *EGFR* L858R is working. Tumor mutation burden (TMB) was investigated by utilizing our panel and showed that *EGFR* L858R had the lowest TMB compared with other *EGFR* mutant subtypes. In addition, analysis of 22 immune cell types from The Cancer Genome Atlas (TCGA) data showed *EGFR* L858R was correlated with low level of CD8 T cells, activated CD4 memory T cells and elevated level of macrophage M2 suggesting an inhibited tumor microenvironment (TME).

**Conclusion:**

Our study identified that *EGFR* L858R neoantigen had the potential to generate cancer vaccines in NSCLC patients with HLA A*33:03. The neoantigen-based vaccines may become an effective salvage regimen for *EGFR* L858R subgroup after targeted therapy or immune checkpoint inhibitors (ICIs) failure.

## Introduction

Lung cancer is still the most common malignancy with morbidity and mortality both ranking first worldwide, and non-small-cell lung cancer (NSCLC) is a subset of lung cancer that has extensive clinical and molecular heterogeneity (1, 2). Epidermal growth factor receptor (*EGFR*) mutations are the most common driver genes in NSCLC, followed by RAS and ALK (3, 4). Only a subset of patients initially responds to targeted therapy, nonetheless, the majority inevitably acquire drug resistance (5–7).

Currently, immune checkpoint inhibitors (ICIs) have achieved positive laboratory results and remarkable clinical responses in the treatment of many kinds of cancer, including NSCLC (8–16). However, in the NSCLC clinical trials, *EGFR* mutant patients benefit less from ICIs than patients with KARS, BRAF, and MET mutations (8, 17–19). Previous studies have reported that antigen expression and presentation deficiency, the low mutation burden, immunosuppressive microenvironment, and upregulation of PD-L1 may be the mechanisms that limited efficacy of ICIs in *EGFR* mutant NSCLC patients (2, 20–23).

Yet, some NSCLC patients whose tumors are harboring *EGFR* mutations do respond to ICIs and studies have continued to focused on the tumor immune phenotype or somatic mutation features to develop novel and more effective treatments for this population. To date, the strategy that utilizes individualized neoantigen vaccines derived from mutated genes against cancers has achieved success in both mouse models and the clinical settings (7). Neoantigens generated from tumor-specific somatic mutations are the optimal targets for T-cells and are capable of mobilizing strong antitumor immune responses (24, 25).

To develop a vaccine that targets individualized neoantigen in NSCLC patients with *EGFR* mutations who do respond to ICIs, we performed a retrospective analysis of 1862 Chinese NSCLC tumor tissues matched with normal tissue samples which were previously profiled using our 1021-gene panel. We then assessed the expression of mutated alleles and predicted possible neoantigens. In this research, we have found that an *EGFR* L858R mutation could be a good target for the development of an individual vaccine for NSCLC patients with HLA A*33:03. We then presented a further investigation on immunological features (HLA LOH, B2M, TMB, and TME) of *EGFR* mutant subtypes to procure the evidence supporting the feasibility of *EGFR* L858R neoantigen. Our results not only provide useful information for predicting response to ICIs, but also introduce a promising treatment for Chinese NSCLC patients with *EGFR* mutations who were failed ICIs therapy and are without alternative therapy.

## Materials and methods

### Cohort

Clinical information of patients was collected from our records. Patients who were diagnosed with NSCLC and underwent targeted sequencing with a 1021-gene panel at Geneplus-Beijing (Beijing, China) were deemed eligible for analysis. For each patient tumor and normal (peripheral blood or normal tissue) samples were available. This study was approved by the Ethics Committee of Fujian Cancer Hospital. Written, informed consent was obtained from all participants before inclusion.

### HLA typing

HLA typing was done using the OptiType v1.0 to obtain the four-digit HLA type at each locus of a patient (26). The Allele Frequency Net Database was utilized to retrieve the allele frequency (AF) of alleles in general Chinese Han populations and carrier frequencies were calculated according to this equation: carrier frequency = 1-(1-AF)^2^.

### Neoantigen prediction and prioritization

For each patient, manually curated somatic mutations (missense or in-frame indel, AF≥0.05) in coding regions were also retrieved from previous records in our database. Neoantigens were predicted using netMHCpan v4.0 (27). Candidates with IC50 mut <500 nM and IC50 wild >=500 nM were considered for further analysis. A putative neoantigen was considered mutant-specific if the IC50 mut is <500 nM, and especially, it is considered as a “strong binder” if the IC50 mut is <50 nM.

### Loss of Heterozygosity (LOH) in HLA genes

The LOH status at all three human leukocyte antigen (HLA) loci was inferred using the lohhla algorithm developed by McGranahan et al. (28). A locus was considered impacted by LOH if the computed *p*-value (‘PVal_unique’ in the output) was <0.01. A patient with a LOH at an HLA locus was defined as one who had at least one HLA locus impacted by LOH. All other patients (including those who have homozygous alleles at all three HLA loci) were considered not affected by HLA LOH.

### Mutation number across four *EGFR* mutation type

Samples were categorized into four sub-groups: with L858R mutation, with deletions in exon 19 (19del), with other *EGFR* mutations, and *EGFR* wild types (WT). Mutations in each sample were quantified by counting the total number of non-synonymous ones. Group-wise Kruskal-Wallis tests were then performed.

### NSCLC datasets and preprocessing in TCGA

Somatic mutations and RNA-sequencing (RNA-seq) data were downloaded from TCGA (https://portal.gdc.cancer.gov/). In consideration of no *EGFR* L858R mutation was found in 495 lung squamous cell carcinoma, therefore, we only mined mutation data from lung adenocarcinoma samples. The lung adenocarcinoma cohort was divided into four clusters as *EGFR* L858R (n=21), EGFR 19del (n=21), EGFR other (n=29) and EGFR WT (n=490). TCGA-LUAD (lung adenocarcinoma) FPKM data containing 594 cancer tissue samples were obtained. After exclusion, analysis was performed on a dataset of 513 lung adenocarcinoma patients who have EGFR mutation status data: *EGFR* L858R (n=21), *EGFR* 19del (n=19), *EGFR* other (n=28), *EGFR* WT (n=445).

### Inference of infiltrating cells in TME

The CIBERSORT (http://cibersort.stanford.edu/) is an analytical tool developed by Newman et al. (29). To quantify the proportions of immune cells in tissue samples. We used the CIBERSORT algorithm and the LM22 gene signature, which was used to distinguish 22 immune cell phenotypes, including B-cells, T-cells, natural killer cells, macrophages, DCs, and myeloid subsets. We utilized CIBERSORT to estimate the fractions of 22 immune cell types among different *EGFR* mutant subtype.

### Statistical analysis

*P* < 0.05 was considered statistically significant. All data were processed using the R software (version 3.6.0), GraphPad 7.0, and AdobeIllustratorCS6.

## Results

### Diversity and prevalence of HLA class I alleles

Data was collected from 1,862 patients who had been diagnosed with NSCLC and underwent targeted sequencing with a 1021-gene panel.

**Figure 1:**
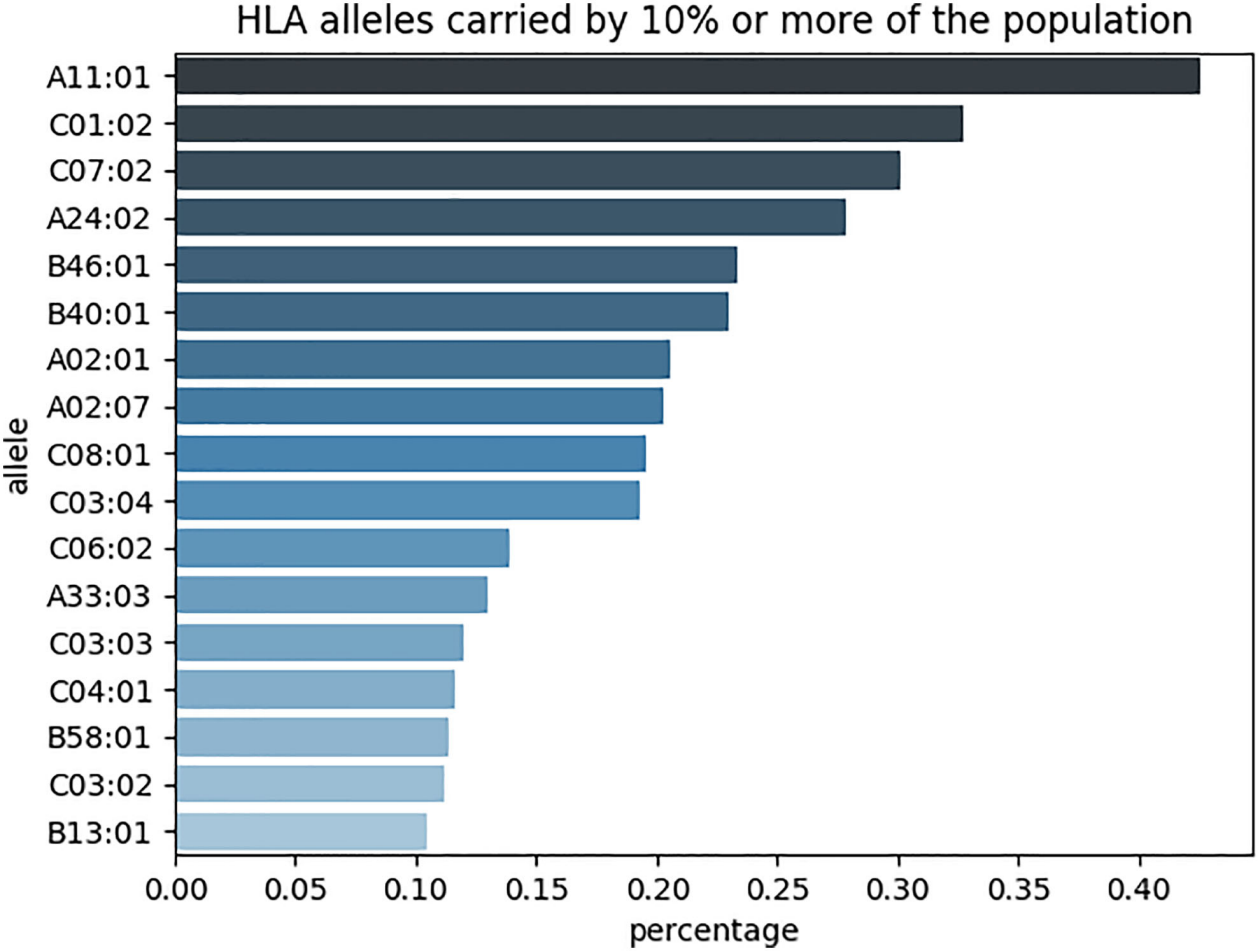
17 different HLA class I alleles (HLA-A, HLA-B, and HLA-C) were found in more than 10% of all patients (n=1862).

We recovered the HLA class I alleles (HLA-A, HLA-B, and HLA-C) for each patient from NGS data. We found 172 different alleles, of which 17 were carried by more than 10% of all patients (**Figure 1**). The most prevalent allele, A*11:01, was found in over 40% of all patients. HLA*11:01 and HLA-A*33:03 allele frequency (AF) are roughly comparable to the numbers retrieved from the Allele Frequency Net Database (30) (**Supplementary Tables 1**, **2**). A rarefaction curve shows that the selected patients covered a large portion of HLA alleles, although it did not reach saturation (**Supplementary Figure 1**). Therefore, the selected population was not biased towards certain allele types and the reported carrier rates were reliable.

### Recurrence of genes and mutations

**Figure 2:**
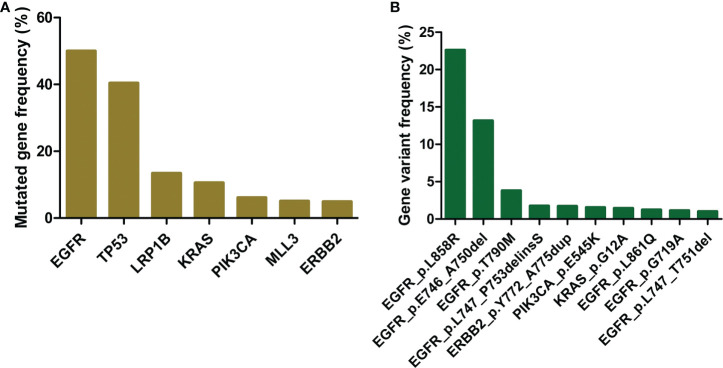
Recurrence of genes and mutations. **(A)** Mutated gene frequency among all patients. **(B)** Gene variant frequency among all patients.

Somatic missense or in-frame indel mutations were selected with an AF greater than or equal to 0.05 for neoantigen prediction and post-prediction analysis. We detected over 10,000 mutations across all patients (about 5 per patient). These mutations affected more than 800 genes. Genes *EGFR* and TP53 were the most frequently mutated genes, they were found mutated in 50% and 40% of all patients, respectively. They were followed by LRP1B and KRAS, which were mutated in 13% and 11% of all patients, respectively (**Figure 2A**). When inspected at variant level, *EGFR* mutations L858R and E746_A750del were overwhelmingly dominant. The frequencies were 23% and 13% for each, over 7-fold and 4-fold higher than the third mutation on the list. The *EGFR* genotyping results mainly agreed with previous studies in the prevalence of driver mutations in NSCLC patients (3, 4, 31). Interestingly, LRP1B mutations were not among the top at variant level (highest frequency at 0.11%), although the gene was mutated in a moderate proportion of patients (**Figure 2B**). Despite the aforementioned genes and mutations, a large majority of these genes and mutations were carried by few patients, typically less than 1% of the population.

### Overview of putative neoantigens

All 8- to 11-mer peptides were derived from all selected mutations and predicted their binding affinity to the patient’s HLA class I complexes to identify patient-specific neoantigens. The prediction was performed for both mutant (MT) and wild type (WT) peptides. We considered a MT peptide a candidate neoantigen if the IC50 MT is smaller than 500 nM and the corresponding IC50 WT is greater than or equal to 500 nM. Furthermore, we categorized candidate neoantigens into “strong binder” and “weak binder” groups by the IC50 MT threshold of 50 nM.

**Figure 3:**
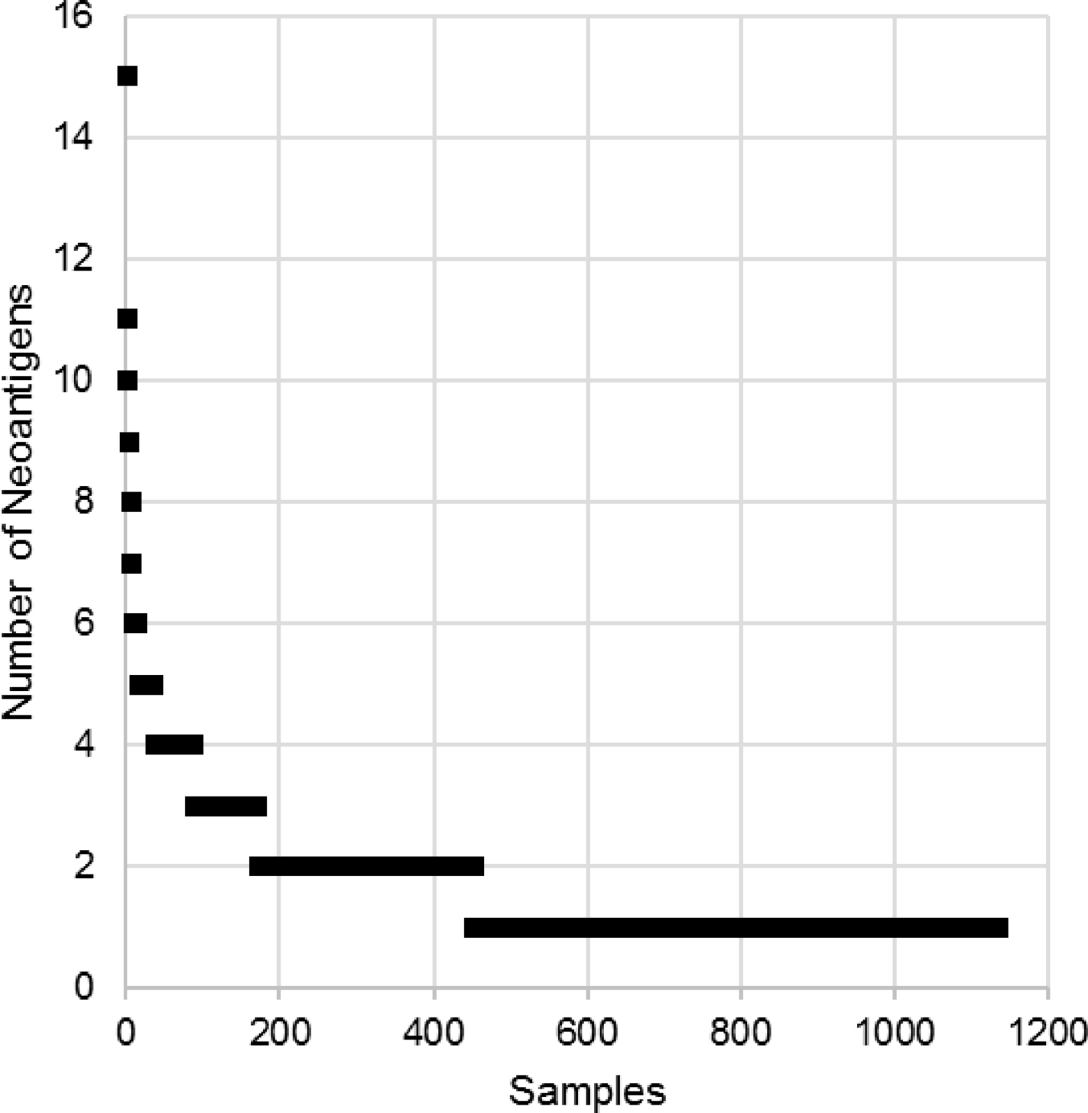
The number of neoantigens in each sample.

**Figure 4:**
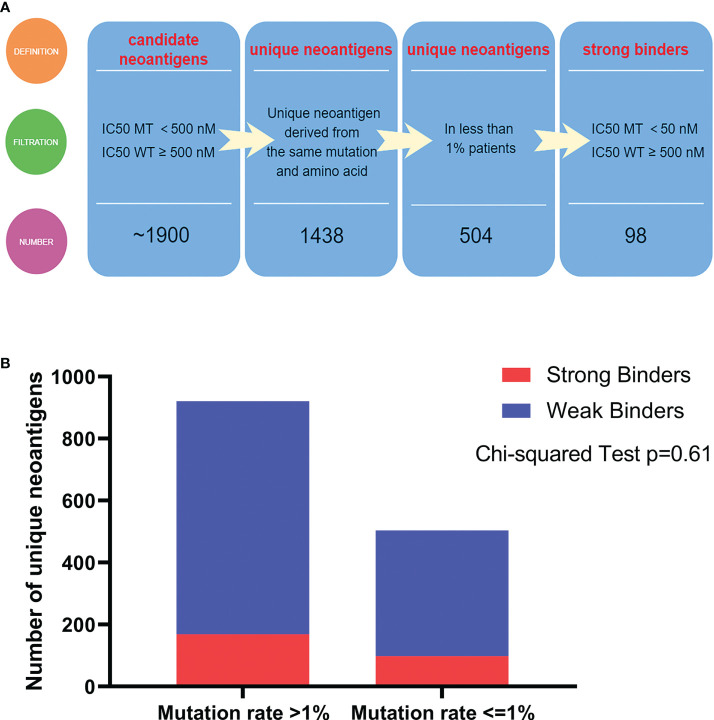
Overview of putative neoantigens. **(A)** Analytic pipelines to output putative neoantigens. **(B)** The proportions of strong and weak binders in two groups. The two groups were categorized by the mutation rate >1% samples vs. <=1% samples.

We identified ~1900 candidate neoantigens in approximately 60% of all patients (1122/1862). The number of neoantigen ranged from 1 to 15, with a median of 1. More than half of the patients were predicted to possess only one neoantigen (**Figure 3**). We further shortened the list to 1438 unique neoantigens. A neoantigen was deemed “unique” if there was no other neoantigens derived from the same mutation and of the same amino acid sequence. About 35% of these unique neoantigens (504/1438) were derived from mutations found in less than 1% of patients. And among these, 19% (98/504) were strong binders (**Figure 4A**). Of the 934 unique neoantigens derived from frequently mutated genes (genes that were found mutated in more than 1% of all samples), 18% (169/934) were strong binders. Also, there were more than 1% of neoantigens (13/934) with ambiguous binding strength, as they were able to bind different HLA molecules with varying affinities. The neoantigens were further categorized into two groups by the mutation rate of the related genes (>1% samples vs. <=1% samples). No statistically significant difference was found between the proportions of strong and weak binders in the two groups (Chi-square Test, p = 0.61) (**Figure 4B**). The result indicates that concurrently mutated genes do not relate with increased proportion of strong binding neoantigens.

### Quantifying neoantigens

The “neoantigen frequency” was calculated, defined in this study as the number of neoantigens related to a gene divided by the number of all mutations targeting this gene, for each neoantigen-producing gene (32). We did this from two perspectives: (1) the total neoantigen frequency, which reflects the “ability” of a gene to produce neoantigen, and (2) the neoantigen frequency corresponding to a specific HLA allele. Note that the latter might be larger than the former, because when calculating the total neoantigen frequency, neoantigens that were able to bind more than one HLA molecule were only counted once. Genes mutated in less than 1% of all patients were removed. By doing this, we also removed any genes targeted by less than 10 mutations. The *EGFR*, with a neoantigen frequency of 0.646, was the top one neoantigen producing gene, and most of these neoantigens were predicted to bind to A*11:01 (allele-specific neoantigen frequency 0.150). We noticed that some genes that were not so prevalent across patients still exhibited a high neoantigen frequency, like ERBB2 (0.563; mutated in 4.9% of patients), CTNNB1 (0.321; 3.2% of patients), and BRAF (0.357; 3.0% of patients) (Data not shown).

**Figure 5:**
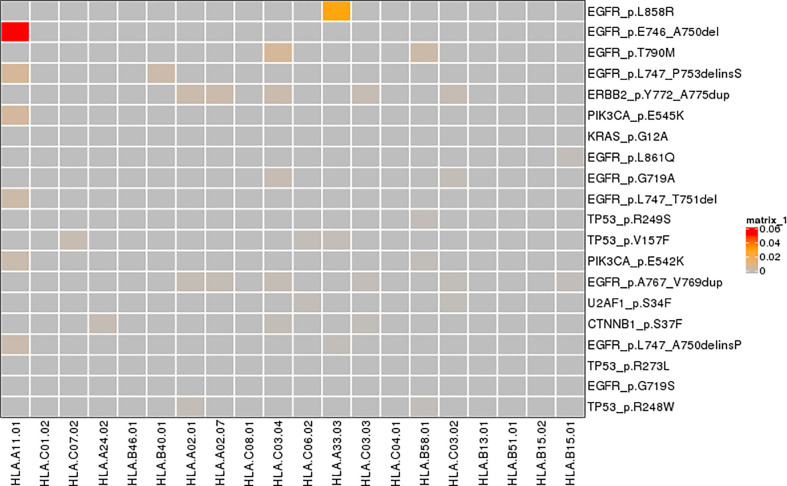
Neoantigens derived from a mutation frequency corresponding to a specific HLA allele.

We repeated the above procedures at variant level. Instead of calculating frequency, we counted the number of neoantigens derived from a mutation directly, in total or HLA-specific. The number of per-mutation neoantigens ranged from 1 to 5. The *EGFR* L858R, the most prevalent mutation, produced four neoantigens. Two mutations were strong binders, while most mutations (except TP53 R110L, which was found in only 0.64% of all patients) produced no more than one strong binder (**Figure 5**).

### Finding shared neoantigens

To find shared neoantigens that could be a good target for generalized neoantigen-based vaccines we further investigated the top two mutations by frequency; *EGFR* L858R and E746_A750del. The two strong binders derived from *EGFR* L858R were HVKITDFGR and RAKLLGAEEK. The latter binds to A*30:01 (IC50 MT = 47.5 nM and IC50 WT = 881.9 nM). Peptide HVKITDFGR binds to three HLA complexes; A*31:01 (IC50 MT = 18.9 nM and IC50 WT = 11653.7 nM), A*33:03 (IC50 MT = 22.9 nM and IC50 WT = 12734.0 nM), and A*68:01 (IC50 MT = 19.6 nM and IC50 WT = 8625.5 nM). The shared frequency of L858R and A*33:03 is 2.93% (22.61% × 12.94%).For the other two the percentages were 1.19% (A*31:01, 22.61% × 5.26%) and 0.28% (A*68:01, 22.61% × 1.24%). The most shared combination is E746_A750del and A*11:01 with a frequency of 5.60%. However, neither of the two neoantigens derived from this mutation was a strong binder (**Table 1**).

**Table 1:** Shared neoantigens based on *EGFR* L858R and E746_A750del.

EGFR mutation	EGFR neoepitope	HLA restriction	IC50 MT (nM)	IC50 WT (nM)	shared frequency (%)
L858R	HVKITDFGR	A*31:01	18.9	11653.7	1.19
		A*33:03	22.9	12734.0	2.93
		A*68:01	19.6	8625.5	0.28
	RAKLLGAEEK	A*30:01	47.5	881.9	1.71
E746_A750del	IPVAIKTSPK	A*11:01	158.2	31132.7	5.60
		A*03:01	70.7	30763.0	0.63
		A*03:02	376.4	29825.9	0.05
		A*11:02	158.2	31132.7	0.48
		A*11:20	65.1	26653.4	0.03
		A*68:01	429.6	11669.5	0.16
	AIKTSPKANK	A*30:01	355.0	5721.5	1.00

### Prevalence of HLA LOH across NSCLC

**Figure 6:**
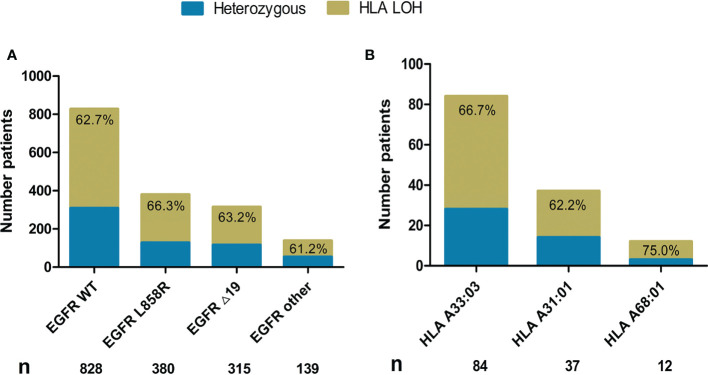
Frequency of HLA LOH in NSCLC. **(A)** The HLA LOH rate in the *EGFR* WT (n=828), *EGFR* L858R (n=380), *EGFR* 19del (n=315), and *EGFR* other (n=139). **(B)** The HLA LOH rate in HLA A*33:03 (n=84), HLA A*31:01 (n=37), and HLA A*68:01 (n=12).

In order to predict the ability to present neoantigens of different *EGFR* mutant subtypes (*EGFR* L858R*, EGFR* 19del*, EGFR* other rare*, EGFR* WT), we identified HLA LOH in our cohort. We analyzed 1731 tumor exomes and found 639 patients (36.9%) who were heterozygous at all HLA-I loci and 1092 patients (63.1%) who had LOH in at least one HLA-I locus in tumors in total (**Supplementary Table 3**). The HLA LOH occurrence rate was higher than the 40% reported in a previous study (28). HLA LOH was calculated for the *EGFR* WT (n=828) and *EGFR* mutant tumors harboring *EGFR* L858R (n=380), *EGFR* 19del (n=315), and *EGFR* other (n=139) (**Figure 6A**). We did not find that HLA LOH had any association with *EGFR* mutation status. Additionally, we examined the HLA LOH of selected HLA (A*33:03, A*31:01, and A*68:01) and also found no difference (**Figure 6B**).

Some mutations that are vital for antigen presentation and MHC class I expression were detected. In our cohort of 1862 NSCLC patients, only seven tumors were found to harbor β2-microglobulin (B2M) mutations and there was no difference among different *EGFR* mutant subtypes (**Supplementary Table 4**). No further mutations like TAP1, TAP2, LMP2 and LMP7 were identified in our cohort (**Supplementary Table 5**).

### Association between *EGFR* mutant subtypes and mutation number

**Figure 7:**
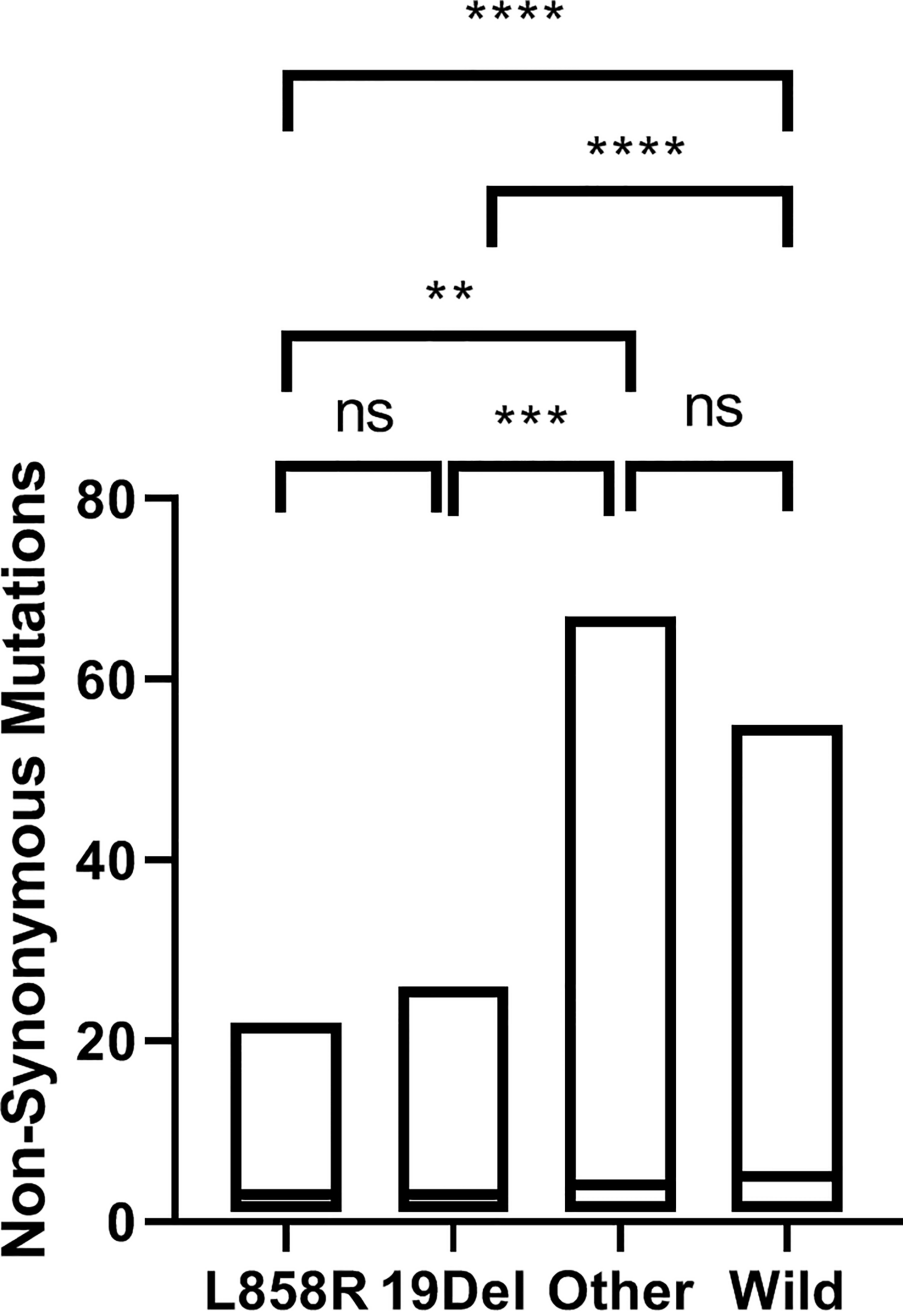
Non-synonymous mutations in NSCLC from our cohorts. The median non-synonymous mutations of *EGFR* L858R (n=421) was three, of *EGFR* 19del (n=367) was three, of *EGFR* other (n=145) was four and of *EGFR* WT (n=604) was five. **p < 0.01; ***p < 0.001; ****p < 0.0001 , ns, no significance.

To examine whether the *EGFR* mutant status influenced the tumor mutation number, we determined the mutation numbers across *EGFR* mutation subtypes in NSCLC tumors from our cohorts. The median of *EGFR* WT (n=604) was five non-synonymous mutations, *EGFR* L858R (n=421) was three non-synonymous mutations, *EGFR* 19del (n=367) was three non-synonymous mutations, and *EGFR* other (n=145) was four non-synonymous mutations. The mutation number was significantly lower in *EGFR* L858R and *EGFR* 19del tumors compared with *EGFR* other and *EGFR* WT tumors. There was no difference between *EGFR* L858R and *EGFR* 19del, which was different from the previous report that *EGFR* 19del mutant lung cancers had a lower mutation number compared with *EGFR* L858R mutant lung cancers (2, 20) (**Figure 7**).

### Association between *EGFR* mutant subtypes and immune infiltration

**Figure 8:**
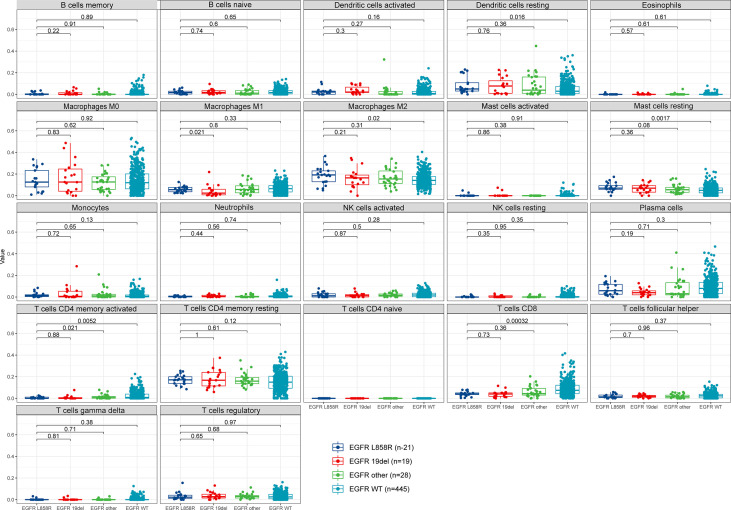
TME in NSCLC from TCGA database. TME, tumor microenvironment. TCGA, The Cancer Genome Atlas.

When looking at the difference of 22 immune cells in *EGFR* mutant subtypes, *EGFR* L858R mutation were found to be associated with the relatively low level of CD8 T cells (*P*=0.00032), activated CD4 memory T cells signatures (*P*=0.0052) and elevated level of macrophage M2 (*P*=0.02) compared to *EGFR* WT tumors. However, the differences were not significant among *EGFR* L858R, *EGFR* 19del and *EGFR* other sites mutations (**Figure 8**).

## Discussion

NSCLC accounts for about 85% of all lung cancers and is a tumor with a high mutational load (33). Although NSCLC harbors many known driven mutations, the inter-individual genomic heterogeneity is extensive. Distinct molecular subtypes differ in sensitivity to various treatments (2). For instance, for treating *EGFR*-driven lung cancers, *EGFR* TKIs has been the first choice. However, the acquired resistance to TKIs is inevitable (5, 6, 34). As an emerging therapeutic approach with the potential for durable responses, ICIs are not recommended for *EGFR*-driven lung patients due to less benefit derived compared with other molecular subgroups (8, 17, 18, 35). However, an *EGFR* mutation is the most common gene alteration in NSCLC. There is a considerable need to find an effective treatment option to significantly increase immunotherapy efficacy in this subgroup.

In this study, we explored neoantigens in 1862 Chinese NSCLC patients who underwent targeted sequencing with a 1021-gene panel. Even though some of the same mutations were shared among different patients, not every mutation would play a role as a neoantigen as the binding affinity to each patient’s own HLA might vary (7, 36, 37). By combining the shared frequency and binding affinity to identify tumor specific somatic mutations, our data revealed that *EGFR* L858R was the top neoantigen producing gene allele, and most of these neoantigens were predicted to bind to A*33:03. We then presented a further analysis on immunological features of *EGFR* mutant subtypes to procure the evidence supporting the feasibility of *EGFR* L858R neoantigen.

A key step in neoantigen presentation and recognition by T-cell receptors is controlled by HLA. Hence, not only the binding affinity of the peptide to the HLA but also loss of HLA expression, which is caused by HLA haplotype loss or mutation of antigen presentation machinery genes such as B2M, needs to be taken into account (28, 38–40). We found that HLA LOH occurred in 63.1% NSCLC in our cohort, higher than 40% reported in a previous study and had no significant association with *EGFR* mutant subtypes (28). Furthermore, the HLA LOH of selected HLA (A*33:03, A*31:01, and A*68:01) also did not show any difference. We next examined B2M aberrations. Specifically, we found only one form of frameshift mutation in B2M: p.L15Ffs*41 and did not find B2M aberrations to be significantly enriched in any subtypes of *EGFR* mutations. Since B2M is essential to the assembly of all HLA class I complexes (41), and HLA LOH may facilitate immune evasion (38), our negative findings indicated that the *EGFR* L858R may not have a deficiency in neoantigen presentation, at least HLA LOH and B2M mutations did not play a crucial role in the immune resistance of *EGFR* L858R patients.

TMB contributes to enhancing antigenic response through the generation of neoantigens (42, 43). Accordingly, we next sought to evaluate the correlation between the attributes of the TMB and *EGFR* mutant subtypes using our panal. Our panel analyses demonstrated that *EGFR* L858R and *EGFR* 19del had the lowest TMB compared to other *EGFR* rare sites mutants and *EGFR* WT, though no difference was noted in *EGFR* L858R and *EGFR* 19del. This is in line with the lower response rate of *EGFR* mutant NSCLCs treated with ICIs, for which low TMB was deemed to be a major culprit of low efficiency of immunotherapy for *EGFR* L858R NSCLC. However, this was different from other studies that *EGFR* 19del mutant lung cancers had a lower TMB compared with *EGFR* L858R mutant lung cancers (2, 20), might be due to, for instance, different races, histology and stages. Moreover, tumor cells are embedded in the tumor microenvironment (TME), suggesting that intercellular relationships are as important as genomic factors (44, 45). In our study, we estimated the fractions of 22 immune cell types of NSCLC from TCGA and studied the correlation between the TME and *EGFR* mutant subtypes. We found *EGFR* L858R was correlated with lower percentage CD8 cells, lower percentage activated CD4 memory T cells and higher percentage macrophage M2 compared with *EGFR* WT. Taken together, these revealed an inhibited TME in the *EGFR* L858R subgroup.

We assembled the largest cohort of NSCLC cases to explore tumor-specific somatic mutations by targeted sequencing with a 1021-gene panel for developing neoantigen vaccines. In our analysis, the *EGFR* L858R neoantigen was identified in an HLA subtype-specific manner that could be used to generate cancer vaccines in HLA A*33:03 subsets patients. *EGFR* L858R in HLA A*33:03 patients would be relevant to 2.93% of the population. Given that lung cancer is the most common cancer, the percentage of patients who may benefit is considerable. We then proposed that the lower TMB and inhibited TME may be the reason for the week immunogenicity of the *EGFR* L858R subset of NSCLC. There were no deficiencies in the HLA LOH and B2M mechanisms, suggesting that the process of antigen presentation of *EGFR* L858R is working.

Our research has some insufficiency. One limitation was that a 1021-gene panel lacks sufficient sequencing data compared with WES or WGS, and only covers a proportion of all coding regions. With the exception of B2M, it did not cover gene mutations related to the HLA presentation which have been implicated as resistance mechanisms to ICIs, like TAP1, TAP2, LMP2 and LMP7 (40, 46, 47). However, since the panel covers most concurrently mutated genomic regions, it is capable of capturing necessary information. In addition, as an indispensable component of neoantigen peptide recognition, the T-cell receptor (TCR) repertoire profiling needs to be explored (48). Recent work on NSCLC has investigated whether the TCR repertoire enables assessment of T cell diversity and T cell clonal expansion and indicated that *EGFR* mutant tumors exhibits lower T cell clonal expansion (49, 50). In the future, we plan to perform TCR sequencing to elucidate whether there exist significant differences in the TCR repertoire diversity in *EGFR* mutant subtypes, aiming to investigate the distinct characteristics of TCR repertoire patterns in *EGFR* L858R. Another possible limitation of this study is that we lack available sequencing data to directly compare TME in this cohort. To address this, we utilized the TCGA data source, but this data source does not represent the real tumor immunogenomic landscape in our Chinese cohort. At last, this is a retrospective study and the clinical information like stages and treatment strategies were incomplete. So we could not conduct stratified analysis to explore some underlying mechanisms.

We excluded frameshift mutations from analysis. The rationale behind this is that there is a chance to raise false positives. Such mutations often result in premature termination codons, which cause the degradation of transcripts *via* nonsense-mediated mRNA decay (NMD) before translation (51). An approach to assess NMD efficiency through RNA-Seq has been published (52), but is not applicable here due to the lack of RNA-Seq data. Still, we estimated to what extent our findings are biased. A total of 975 frameshift mutations (915 unique) were detected, spanning 670 samples. We recalculated the mutation frequency for each gene with frameshift taken into account. The top 6 genes by mutation frequency did not change, while the remaining genes were reordered. For some genes, the mutation frequency increased after the recalculation, such as *TP53* and *LRP1B*. This indicates that in some patients only frameshift mutations were detected on these genes. *TP53* mutation frequency increased from 40.44% to 48.34%, indicating that we probably have underestimated its potential role in neoantigen producing. However, the most frequent frameshift mutation *STK11* P281Rfs*6 was shared by only six patients, which translated into a percentage of 0.3% (**Supplementary Table 4**). This did not serve our purpose of finding shared neoantigens.

In summary, our research identified that *EGFR* L858R neoantigen had the potential to generate cancer vaccines in NSCLC patients with HLA A*33:03 and revealed the possible underlying immunological features between *EGFR* mutant subtypes. Our finding provides the basis for further investigations into which neoantigen-based vaccines may become an effective treatment strategy for patients with *EGFR* L858R mutation.

## Data availability statement

The original contributions presented in the study are included in the article/**Supplementary Material**. Further inquiries can be directed to the corresponding authors.

## Ethics statement

The studies involving human participants were reviewed and approved by the Ethics Committee of Fujian Cancer Hospital. The patients/participants provided their written informed consent to participate in this study.

## Author contributions

JiL, JuL, S-GH, BL carried out the whole research. X-BZ, J-NX, Y-QZ, XG, Y-TY, XY statistically analyzed all the data and graphed. LC, Y-FH, HL, J-PL help in assays. C-BC, GC and X-WZ developed the concept. X-FW and YC obtained the funds and organized the study. All authors read and approved the final manuscript. All authors contributed to the article and approved the submitted version.

## Funding

The work was supported by Fujian Provincial Clinical Research Center for Cancer Radiotherapy and Immunotherapy (Grant No. 2020Y2012), the National Natural Science Foundation of China (Grant No. U1705282, 32000550), Joint Funds for the Innovation of Science and Technology, Fujian province (Grant No. 2021Y9227) and Startup Fund for scientific research, Fujian Medical University (Grant No. 2019QH1200).

## Acknowledgments

We appreciate all subjects who participated in this study and all colleagues (Cancer Bio-immunotherapy Center, Fujian Cancer Hospital) for technical support.

## Conflict of interest

Authors S-GH, Y-QZ, XG, Y-TY, and XY were employed by company Geneplus.

The remaining authors declare that the research was conducted in the absence of any commercial or financial relationships that could be construed as a potential conflict of interest.

## Publisher’s note

All claims expressed in this article are solely those of the authors and do not necessarily represent those of their affiliated organizations, or those of the publisher, the editors and the reviewers. Any product that may be evaluated in this article, or claim that may be made by its manufacturer, is not guaranteed or endorsed by the publisher.

## Supplementary material

Supplementary Figure 1 The rarefaction curve shows the selected population was not biased towards certain allele types.

## Abbreviations

NSCLC, non-small-cell lung cancer; ICIs, immune checkpoint inhibitors; EGFR, Epidermal growth factor receptor; 19 del, deletions in exon 19; HLA, human leukocyte antigen; HLA LOH, HLA loss of heterozygosity; TMB, Tumor mutation burden; TCGA, The Cancer Genome Atlas; TME, tumor microenvironment.
