# Supplementary material for: An EGFR L858R mutation identified in 1862 Chinese NSCLC patients can be a promising neoantigen vaccine therapeutic strategy
Source: Front Immunol. 2022 Nov 23;13:1022598. doi: 10.3389/fimmu.2022.1022598 (PMC9727402; doi:10.3389/fimmu.2022.1022598)
Supplement: Supplementary file 1 [file DataSheet_1.docx]

**Supplementary materials**

**Table S1. Allele frequencies of HLA-A*11:01 in different population**

| **Population** | **Allele frequency** | **Sample size** |
| --- | --- | --- |
| China Yunnan Province Han | 0.3170 | 101 |
| China South Han | 0.2770 | 284 |
| China Canton Han | 0.2670 | 264 |
| China Beijing Shijiazhuang Tianjin Han | 0.2020 | 618 |
| China Jiangsu Province Han | 0.1777 | 334 |
| China Jiangsu Han | 0.1650 | 3238 |

**Table S2. Allele frequencies of HLA-A*33:03 in different population**

| **Population** | **Allele frequency** | **Sample size** |
| --- | --- | --- |
| China Yunnan Province Han | 0.0840 | 101 |
| China South Han | 0.1150 | 284 |
| China Beijing Shijiazhuang Tianjin Han | 0.0620 | 618 |
| China Jiangsu Province Han | 0.1045 | 334 |
| China Jiangsu Han | 0.1118 | 3238 |

**Table S3. Seven tumors harbor β2-microglobulin (B2M) mutations in our cohort and no difference in this seven *EGFR* mutant subtypes**

| **Sample** | **B2M (7/1862)** | | | **EGFR** |
| --- | --- | --- | --- | --- |
|  | **HGVSc** | **HGVSp** | **Function** |  |
| 180008394FD | c.31G>C | p.A11P | missense | WT |
| 180012241FD | c.248_253delATCTCT | p.Y83_L84del | cds-del | WT |
| 180016964FD | c.7C>T | p.R3C | missense | p.E709K  p.L858R |
| 180032355FD | c.5C>G | p.S2C | missense | WT |
| 180011886FD | c.37CT[4>3] | p.L15Ffs*41 | frameshift | p.F55L |
| 180031892FD | c.37CT[4>3] | p.L15Ffs*41 | frameshift | WT |
| 190010746FD | c.37CT[4>3] | p.L15Ffs*41 | frameshift | WT |
