# Supplementary figures and images for: An EGFR L858R mutation identified in 1862 Chinese NSCLC patients can be a promising neoantigen vaccine therapeutic strategy
Source: Front Immunol. 2022 Nov 23;13:1022598. doi: 10.3389/fimmu.2022.1022598 (PMC9727402; doi:10.3389/fimmu.2022.1022598)

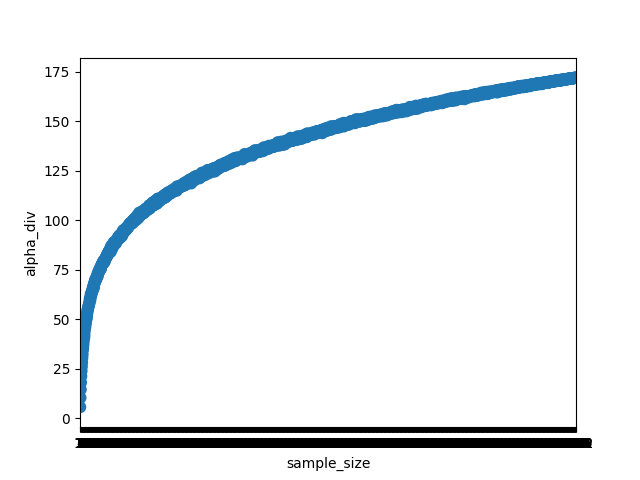

Supplement: Supplementary Figure 1 — The rarefaction curve shows the selected population was not biased towards certain allele types. [file Image_1.tif]
